# 

**Published:** 1892-04-09

**Authors:** 


### CONTENTS.

**More Room, or Better Room?** - - - - - - - - 17

**Passing Topics.**—Mud and Muddle - - - - - - - 18

**The Doctor's Armchair.**— Unmarried Hampstead: Much-Married Whitechapel - - - - - - - - - - 19

**The Story of the Insane from Year to Year** - - - 20

**Annotations.**—The Moral of Mrs. Siddons' "Chops."—The House Surgeon Question.—How the Dumb Speak - - - - - 23

**Notes and News,** 24; **Scraps and Gleanings** - - - 25

**The Practitioner's Mirror and Retrospect—**

Medicine.—Rumination in Human Beings - - - - - 26

Surgery.—The Diagnosis, Localisation, and Surgical Treatment of Tumours Pressing on the Spinal Cord - - - - - - 26

Ophthalmology.—The Abuse of Mercury in the Treatment of Eye Diseases - - - - - - - - - - - - 27

New Drugs, Appliances, and Things Medical.—Sedox Absorbent Dressings - - - - - - - - - - 28

**Medical History from the Earliest Times.—By E. T.** Withington, M.A., M.B. Oxon. III.—Medicine as Practised by Uncivilised Man - - - - - - - - - - - 21

**Everybody's Page** - - - - - - - - - 22

**Drunkenness and Crime** - - - - - - - - 28

**The Institutional Workshop—**

The Chronic Indigence of our Hospitals, with some Suggestions for its Remedy. By B. Burford Rawlings. II.—Difficulties of Hospital Finance - - - - - - - 29

Furniture and Fittings.—Gloucester Asylum - - - - 31

Hospital Construction.—The New Hall for Operations at the Large Municipal Hospital, Copenhagen—The Alice Memorial Hospital, Hong Kong - - - - - - - - - - 32

Practical Points - - - - - - - - - - - 32

**The Student of Medicine.**—Appointments—Vacancies—Notes from the Schools.

### EXTRA SUPPLEMENT.—THE NURSING MIRROR.

**En Passant.**—Good News from South Africa—Short Items—The Registration of Nurses—The Nurses' Co-operation—Nursing Homes and Paying Patients — Radcliffe Infirmary, Oxford — Lincoln Nurses - - - - - - - - - - - vii
**On the Nursing of Children.** VIII.—Observation of Colour, Posture, &c.—**Appointments—The L.O.S. Examination** viii
**Significant!—For Reading to the Sick.**—The Helping Hand ix
**Notes from Australia** - - - - - - - - - x
**Nursing Uniforms.** IV.—London Hospital - - - - - xi
**News from Jamaica—Notes and Queries** - - - - - xi
**Everybody's Opinion**—Holiday Experiences - - - - xi
**Off Duty.**—A Troublesome Case—**Where to Go** - - - - xii

